# Vitamin-D2 treatment-associated decrease in 25(OH)D3 level is a reciprocal phenomenon: a randomized controlled trial

**DOI:** 10.1186/s12902-019-0337-8

**Published:** 2019-01-18

**Authors:** Muhammad M. Hammami, Kafa Abuhdeeb, Safa Hammami, Ahmed Yusuf

**Affiliations:** 1 Department King Faisal Specialist Hospital and Research Center, Clinical Studies and Empirical Ethics, P O Box # 3354, Riyadh, 11211 Saudi Arabia; 2 Alfaisal University College of Medicine, Riyadh, Saudi Arabia; 3 St. Mary Medical Center, San Francisco, California USA

**Keywords:** Cholecalciferol treatment, Ergocalciferol treatment, 25-hydroxyvitamin D2 level, 25-hydroxyvitamin D3 level

## Abstract

**Background:**

Vitamin-D2 (D2) treatment has been associated with a decrease in 25-hydroxy (25(OH)) vitamin-D3 (D3) level, suggesting that D3 treatment would be preferred to raise total 25(OH) vitamin-D (D) level. We postulated that D2 treatment-associated decrease in 25(OH)D3 level is related to the increase in 25(OH)D level rather than being D2-specific, and thus there would be a similar D3 treatment-associated decrease in 25(OH)D2 level.

**Methods:**

Fifty volunteers were block-randomized to 50,000 IU D2 or placebo orally once (study-1) and fifty volunteers received 50,000 IU D2 orally once and 4 days later block-randomized to 50,000 IU D3 or placebo orally once (study-2). Interventions were concealed from volunteers and research coordinators and blindly-administered. Serum 25(OH)D2 and 25(OH)D3 levels were blindly-determined at baseline and days 14, 28, 42, and 56, post-randomization by high performance liquid chromatography assay. Results of 97 participants were analyzed. Primary outcome measure was day-28 D2-associated change in 25(OH)D3 level in study-1 and D3-associated change in 25(OH)D2 level in study-2, adjusted for baseline levels.

**Results:**

Mean (95% confidence interval) difference between the active and placebo arms in the decrease in day-28 25(OH)D3 (study-1) and 25(OH)D2 (study-2) levels was 13.2 (9.7 to 16.6) and 9.8 (5.2 to 14.4) nmol/L, respectively. Corresponding differences at day-56 were 10.8 (6.8 to 14.8) and 1.7 (− 7.6 to 11.1) nmol/L, respectively. The difference between the placebo and active arms in area-under-the-curve at day-28 (AUC_28_) and day-56 (AUC_56_) were 262.3 (197.8 to 326.7) and 605.1 (446.3 to 784.0) for 25(OH)D3 (study-1) and 282.2 (111.2 to 453.3) and 431.2 (179.3 to 683.2) nmol.d/L for 25(OH)D2 (study-2), respectively. There were significant correlations between day-28 changes in 25(OH)D2 and 25(OH)D3 levels in study-1 (rho = − 0.79, *p* < 0.001) and study-2 (rho = − 0.36, *p* = 0.01), and between day-28 changes in 25(OH)D2 level and baseline 25(OH)D level in study-2 (rho = − 0.42, *p* = 0.003).

**Conclusions:**

Compared to placebo, D3 treatment is associated with a decrease in 25(OH)D2 level similar in magnitude to D2-treatment associated decrease in 25(OH)D3 level; however, the D3-placebo difference in 25(OH)D2 level is shorter-lasting. Changes in 25(OH)D2 and 25(OH)D3 levels are correlated with each other and with baseline 25 (OH) D levels, suggesting a common regulatory mechanism.

**Trial registration:**

ClinicalTrial.gov identifier: NCT03035084 (registered January 27, 2017).

## Background

Both ergocalciferol (vitamin-D2, D2) and cholecalciferol (vitamin-D3, D3) are commonly and effectively used to prevent and treat vitamin-D deficiency. Their active dihydroxy metabolites, 1,25-dihydroxyvitamin D2 (1,25(OH)_2_D2) and 1,25-dihydroxyvitamin D3 (1,25(OH)_2_D3), are comparable in binding to vitamin-D receptor [1], and their 25-hydroxy (25(OH)) metabolites, 25(OH)D2 and 25(OH)D3, are comparable substrates for kidney 1-alpha hydroxylase [2, 3]. Nevertheless, their relative potency in increasing total 25-hydroxy vitamin-D (25(OH)D), the best marker of vitamin-D status, continues to be controversial [2, 4, 6–13].

D2 and D3 differ in their affinity to hepatic 25-hydroxylase and circulating vitamin-D binding protein, inactivation by 24-hydroxylation, and plasma half-life [9, 12, 14]. Further, despite its effectiveness in raising 25(OH)D2 and 1,25(OH)_2_D2 levels, D2 supplementation has been associated with a decrease in 25(OH)D3 [10, 14–18] and 1,25(OH)_2_D3 [19, 20] levels.

The underlying mechanisms of D2-associated decrease in 25(OH)D3 level have not been clearly identified. We previously noted that the D2-associated decrease in 25(OH)D3 level correlated with the increase in 25(OH)D2 level and baseline 25(OH)D level and that when baseline 25(OH)D2 level was measurable (one case), D3 treatment resulted in a similar decrease in 25(OH)D2 level [10], suggesting that the D2-associated decrease in 25(OH)D3 level may merely reflect a response to increasing 25(OH)D level. This interpretation is consistent with the results of numerous studies [8–10, 18, 21, 22], showing significant negative correlation between baseline 25(OH)D level and response to treatment and non-linear response of 25(OH)D level to increasing doses of vitamin-D [23–25].

We postulated that the D2 treatment-associated decrease in 25(OH)D3 may be related to an increase in total 25(OH)D level rather than being specific to D2 treatment and, therefore, there would be a D3 treatment-associated decrease in 25(OH)D2 level of similar magnitude.

## Methods

### Design

Two randomized, placebo-controlled, blinded trials, comparing the effect of D2 treatment to placebo on 25(OH)D3 level (study-1) and the effect of D3 treatment to placebo on 25(OH)D2 level (study-2).

### Participants

Volunteers were recruited via advertisement throughout the King Faisal Specialist Hospital and Research Center (KFSH&RC) and other medical centers in the City of Riyadh, Saudi Arabia. We enrolled healthy non-pregnant adults (age ≥ 18 years) living in Riyadh area who habitually don’t consume more than one serving of milk daily, don’t take vitamin supplements, and have less than 10 h of sun exposure weekly; don’t suffer from granulomatous, liver, or kidney diseases; don’t take anticonvulsants, barbiturates, or steroids; and have a screening 25(OH)D3 (and total 25(OH)D) level >40 and ≤ 65 nmol/L (study-1) or total 25(OH)D ≥ 20 and <40 nmol/L (study-2). Lifestyle habits data were self-reported and collected through a simple questionnaire. Because of the time lapse between screening and enrolment (mean (SD) 20.1 (9.2), 19.3 (10.7), 25.4 (15.8), and 27.0 (17.0) day for study-1 D2 arm, study-1 placebo arm, study-2 D3 arm and study-2 placebo arm, respectively) and assay inter-run variations, levels at enrolment were outside the above-described ranges in some participants. The study was conducted at King Faisal Specialist Hospital and Research Center (KFSH&RC) from February 2017 through November 2017 after obtaining approval of the KFSH&RC Research Ethics Committee. All participants gave written informed consent and were compensated for time and inconvenience.

### Procedures and interventions

Vitamin-D3 50,000 IU (Hayat Pharmaceutical Industries Co., Amman, Jordan) and vitamin-D2 50,000 IU (Breckenridge Pharmaceutical, Inc., Boca Raton, Florida, USA) softgel capsules were purchased from local pharmacies. Placebo softgel capsules were provided by Jamjoom Pharma (Jeddah, Saudi Arabia).

Participants in study-1 were randomized to a single oral dose of 50,000 IU D2 or placebo. Participants in study-2 were first loaded with a single oral dose of 50,000 IU D2, and 4 days later, randomized to a single oral dose of 50,000 IU D3 or placebo. Interventions were administered in the research clinic after a standardized breakfast. Blood samples were drawn at baseline and at days 14, 28, 42, and 56, post-randomization.

Levels of 25(OH)D2 and 25(OH)D3 were blindly measured by a locally validated reversed-phase high performance liquid chromatography assay (HPLC) [26]. The assay uses gradient elution mode and a photodiode array detector and measures D2, D3, 25(OH)D2, and 25(OH)D3 serum levels simultaneously. The assay was selective and did not detect any interference between D2, D3, 25(OH)D2, and 25(OH)D3 peaks. Serum samples were deproteinized with a mixture of methanol and 2-proponol and extracted with hexane. The intra-assay and inter-assay coefficients of variation were, respectively, ≤6.2% and ≤ 8.6% for 25(OH)D2 and ≤ 9.7% and ≤ 8.6% for 25(OH)D3 (measured at 37.5, 125, and 225 nmol/L). Limits of detection and quantification were, respectively, 5 and 12.5 nmol/L for 25(OH)D2 and 25(OH)D3. D2 and D3 levels (limits of quantification 12.5 nmol/L) were used as indicators of participants’ compliance with study procedures and of study-unrelated exposure to D2 and D3. All the samples of each participant were analyzed in the same assay run.

### Randomization and blinding

Blocked (block size = 2) randomization sequences were generated (by MMH) using an online program (http://www.jerrydallal.com/random/assign.htm) [27]. Assignment was concealed from potential participants and recruiting coordinators. Participants, study coordinators, and 25(OH)D analyst were blinded to randomized assignments.

### Sample size

Our previous study [11] showed that compared to placebo, 50,000 IU D2 is associated with a decrease of about 10 nmol/L in 25(OH)D3 level at day-28 with a SD of 10.5 nmol/L. Therefore, the sample size was estimated to be 50 for each study, based on type 1 error of 0.05, type 2 error of 0.20, an expected difference between active treatment and placebo of 10 nmol/L, a SD of 12, and a drop out of 10%.

### Outcome measures and analysis

Primary outcome measures were day-28, D2-associated change in 25(OH)D3 level in study-1 and D3-associated change in 25(OH)D2 level in study-2, adjusted for baseline levels. Predetermined secondary outcome measures were the corresponding changes at day-56, correlation between the changes in 25(OH)D2 level or 25(OH)D3 level on one hand and baseline total 25(OH)D level on the other, and correlation between the changes in 25(OH)D2 and 25(OH)D3 levels in each study. Ad hoc secondary outcome measures were area-under-the-curve from baseline until day-28 (AUC_28_) or day-56 (AUC_56_) for 25(OH)D2 and 25(OH)D3 levels in study-1 and study-2, respectively. Analyses were performed (by MMH) with IBM SPSS Statistics version 21 software. Two-tailed *p*-values and 95% confidence intervals (CI) are reported. CIs for rho values were calculated after r to z transformation, assuming r is approximated by rho.

## Results

**Fig. 1:**
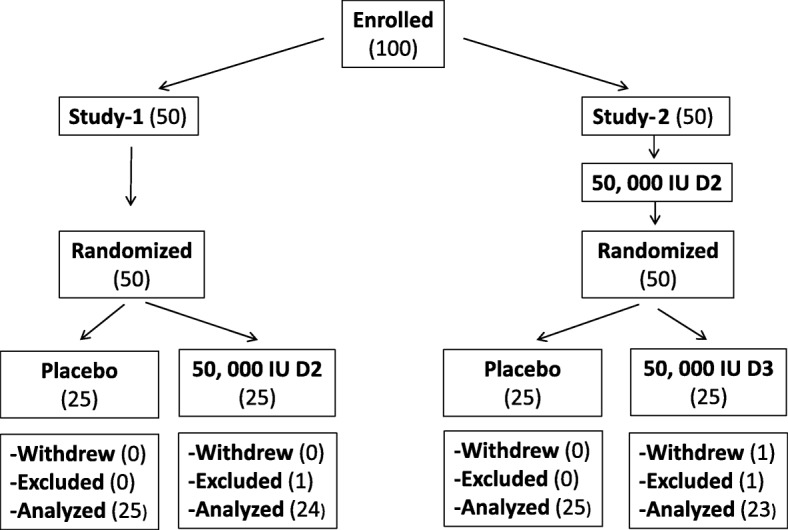
Participants flow chart. One participant was excluded because of formula failure/not ingesting D2 dose (study-1, D2 arm), one withdrew for personal reasons (study-2, D3 arm), and one was excluded because of high 25(OH)D2 before receiving the 50,000 IU D2 loading dose (study-2, D3 arm)

Fifty participants were randomized to either placebo or active intervention in each study; three out of the 100 participants were excluded from analysis **(**Fig. 1). One participant in study-1 (D2 arm) was excluded because blood sample analysis showed no increase in 25(OH)D2 level or D2 level, indicating formulation failure or that the dose was not ingested. In study-2 (D3 arm), one participant withdrew for personal reasons and another was excluded because of high 25(OH)D2 level (83 nmol/L) before receiving the 50,000 IU D2 loading dose, indicating that a large dose of vitamin D was ingested between screening for eligibility and actual enrollment in the study. One participant (study-2, D3 arm) did not have an increase in 25(OH)D2 level in response to D2 loading dose until day-28, and therefore was not included in calculating day-28 change in 25(OH)D2 level. Another participant (study-2, placebo arm) did not have an increase in 25(OH)D2 level in response to D2 loading dose at day zero, and therefore 25(OH)D2 level at day-14 was used as baseline.

Measurable (≥12.5 nmol/L) D2 and D3 levels are generally not expected two weeks after receiving 50,000 IU of D2 or D3 [10]. We therefore used D2 or D3 levels beyond this time frame as indicators of study-unrelated D2 and D3 exposure. In study-1, D2 and D3 levels were, respectively, measurable in 4 and 12% of participants in the D2 arm and 0 and 12% in the placebo arm. In study-2, D2 and D3 levels were, respectively, measurable in 26 and 39% of participants in the D3 arm and 28 and 32% in the placebo arm.

Table 1 summarizes baseline characteristics of the 97 participants who were included in analysis. Mean (SD) age was 33.6 (6.2) year, mean body mass index (BMI was 24.9 (3.9) kg/m^2^, mean habitual sun exposure 1.5 (1.2) hour per week, mean habitual milk intake was 0.4 (0.5) cup per day, and 70% were women. In study-1, mean baseline 25(OH)D3 level was 52.3 (12.5) and 52.4 (12.7) nmol/L in the D2 and placebo arms, respectively. In study-2, mean baseline 25(OH)D2 level (i.e., 4 days after loading with 50,000 IU vitamin-D2) was 39.6 (14.1) and 37.5 (15.5) nmol/L in the D3 and placebo arms, respectively. No adverse events were identified.

**Table 1:** Baseline characteristics of study participants

	Study-1		Study-2^a^		Total
	Vitamin-D2	Placebo	Vitamin-D3	Placebo	
Number	24	25	23	25	97
Age, year	34.4 (5.1)	33.6 (6.3)	34.1 (6.8)	32.4 (6.4)	33.6 (6.2)
Female, number (%)	18 (75)	16 (64)	15 (65)	19 (76)	68 (70)
BMI, kg/m^2^	25.9 (4.5)	24.2 (3.2)	24.7 (3.0)	24.7 (4.4)	24.9 (3.9)
Sun exposure, hour/week	2.0 (1.3)	1.5 (1.3)	1.3 (0.9)	1.3 (1.1)	1.5 (1.2)
Milk intake, cup/day	0.6 (0.5)	0.5 (0.5)	0.3 (0.4)	0.3 (0.4)	0.4 (0.5)
Serum 25(OH)D2, nmol/L	UQ	UQ	39.6 (14.1)	37.5 (15.5)	19.3 (21.8)
Serum 25(OH)D3, nmol/L	52.3 (12.5)	52.4 (12.7)	45.4 (15.6)	45.2 (17.3)	48.8 (15.1)

^a^Received 50,000 IU of vitamin-D2 orally 4 days before obtaining baseline level. Data are means (SD), except as indicated. BMI, body mass index. 25(OH)D2, 25-hydroxyvitamin D2, 25(OH)D3, 25-hydroxyvitamin D3, 25(OH)D, total 25-hydroxyvitamin D. UQ, levels were undetectable in all participants of study-1 except for 2 participants in the vitamin-D2 arm where they were detectable but not quantifiable

### D2-associated decline in 25(OH)D3 level (study-1)

**Fig. 2:**
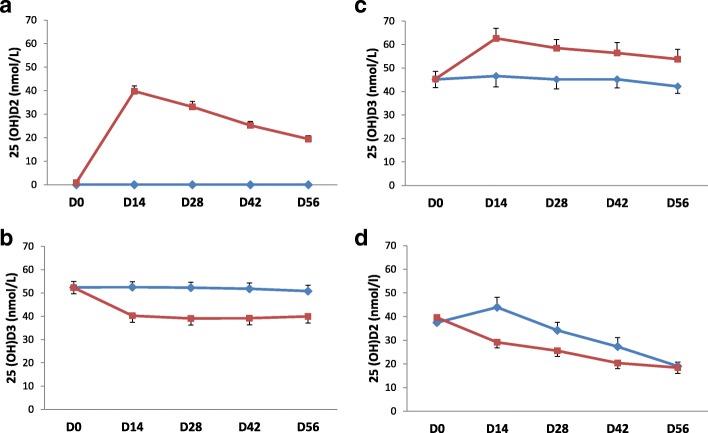
Changes in 25(OH)D2 and 25(OH)D3 levels over 56 days. Data represent mean levels. Error bars represent standard errors of the mean. **a** and **b**, study-1: participants were randomized to a single oral dose of 50,000 IU D2 (red squares) or placebo (blue diamond). **c** and **d**, study-2: participants were randomized to a single oral dose of 50,000 IU D3 (red squares) or placebo (blue diamond) four days after receiving a single oral dose of 50,000 IU D2. 25(OH)D2, 25-hydroxyvitamin D2. 25(OH)D3, 25-hydroxyvitamin D3. D0, D14, D28, D42, and D56 are day of randomization, and 14, 28, 42, and 56 days after randomization, respectively

As shown in Fig. 2a, the administration of a single oral dose of 50,000 IU D2 resulted in marked increase in 25(OH)D2 level that peaked to a mean (SD) of 39.8 (11.0) nmol/L at day-14 and then declined by about 50% to 19.4 (6.9) nmol/L at day-56. There was no change in mean 25(OH)D2 level in the placebo arm (remained undetectable). In the D2 arm, mean 25(OH)D3 level declined from 52.3 (12.5) nmol/L at day zero to 40.2 (13.9) nmol/L at day-14 and then remained essentially the same until day-56 (39.9 (14.0) nmol/L). In contrast, there was little change (52.4 (12.7), 52.5 (11.8), and 50.8 (12.3) nmol/L at days zero, 14, and 56, respectively) in mean 25(OH)D3 level in the placebo arm **(**Fig. 2b).

**Fig. 3:**
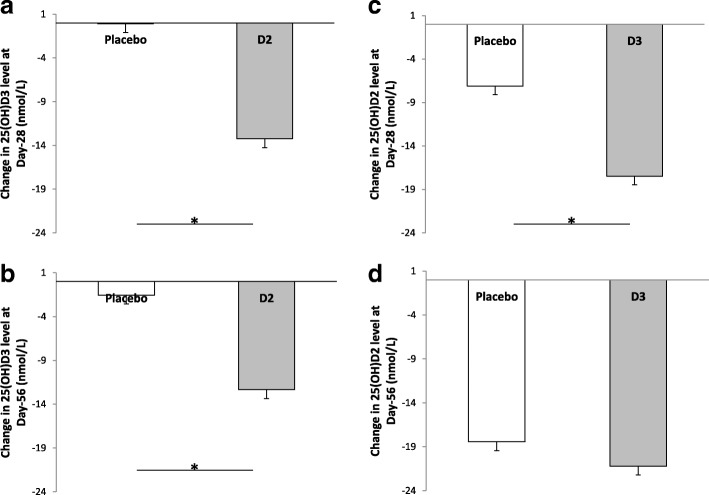
Decrease in 25(OH)D2 and 25(OH)D3 levels from randomization to day-28 (**a** and **c**) or day-56 (**b** and **d**). Bars represent means and standard errors. a and b, study-1: participants were randomized to a single oral dose of 50,000 IU D2 (solid bars) or placebo (open bars). **c** and **d**, study-2: participants were randomized to a single oral dose of 50,000 IU D3 (solid bars) or placebo (open bars) four days after receiving a single oral dose of 50,000 IU D2. 25(OH)D2, 25-hydroxyvitamin D2. 25(OH)D3, 25-hydroxyvitamin D3. *, *p* < 0.001

As shown in Fig. 3a**,** the decline in 25(OH)D3 level in the D2 arm at day-28 (13.3 (6.7) nmol/L) was significantly different from the observed decline in the placebo arm (0.1 (5.6) nmol/L) with an adjusted mean difference of 13.2 (95% confidence interval CI (CI), 9.7 to 16.6) nmol/L, *p* < 0.001. The corresponding adjusted mean difference at day-56 was 10.8 (CI, 6.8 to 14.8) nmol/L, *p* < 0.001 (Fig. 3b)**.**

**Fig. 4:**
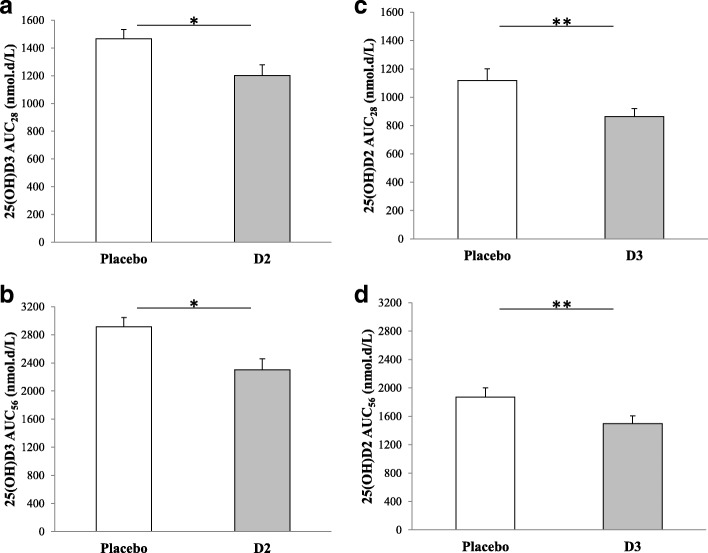
25(OH)D2 and 25 (OH) D3 area-under-the-curves from randomization to day-28 (AUC_28_) or day-56 (AUC_56_). Bars represent means and standard errors. **a** and **b**, study-1: participants were randomized to a single oral dose of 50,000 IU D2 (solid bars) or placebo (open bars). **c** and **d**, study-2: participants were randomized to a single oral dose of 50,000 IU D3 (solid bars) or placebo (open bars) four days after receiving a single oral dose of 50,000 IU D2. 25(OH)D2, 25-hydroxyvitamin D2. 25(OH)D3, 25-hydroxyvitamin D3. *, *p* < 0.001. **, *p* < 0.005

Figure 4 shows 25(OH)D3 AUC_28_ and AUC_56_ in the placebo and D2 arms. Mean 25(OH)D3 AUC_28_ was 1467.3 (334.9) and 1202.1 (375.2) nmol.d/L in the placebo and D2 arms, respectively, with a significant adjusted mean difference of 262.3 (CI, 197.8 to 326.7) nmol.hr./L, *p* < 0.001 (Fig. 4a).The corresponding adjusted mean difference at day-56 was 605.1 (CI, 446.3 to 784.0) nmol.hr./L, *p* < 0.001 (Fig. 4b)**.** Compared to placebo, 25(OH)D3 AUC_28_ and AUC_56_ were reduced by 18 and 21%, respectively.

**Fig. 5:**
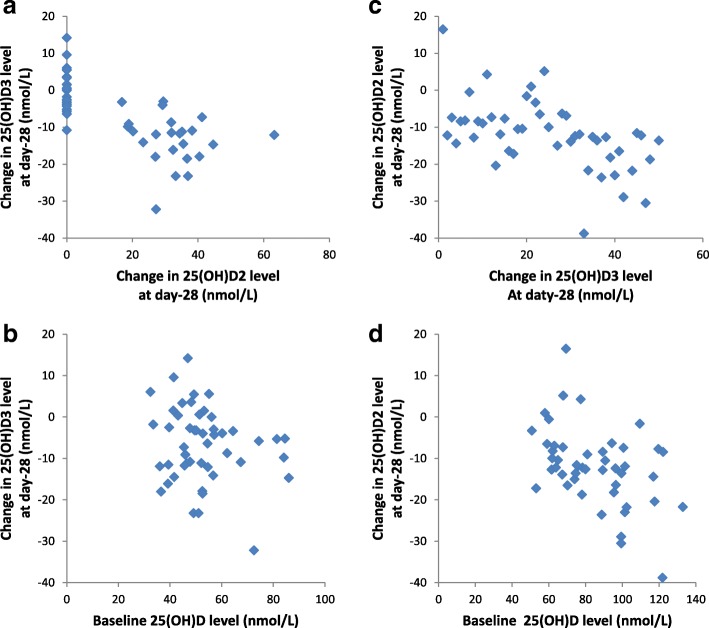
Correlation between changes in day-28 25(OH)D2 and 25(OH)D3 levels and baseline total 25(OH)D level. **a** and **b**, study-1: participants were randomized to a single oral dose of 50,000 IU D2 or placebo. **c** and **d**, study-2: participants were randomized to a single oral dose of 50,000 IU D3 or placebo four days after receiving a single oral dose of 50,000 IU D2. 25(OH)D2, 25-hydroxyvitamin D2. 25(OH)D3, 25-hydroxyvitamin D3. 25(OH)D, total 25-hydroxyvitamin D. a, rho = − 0.79 (CI, − 0.66 to − 0.88), p < 0.001. b, rho = − 0.17 (CI, 0.12 to − 0.43), *p* = 0.31. c, rho = − 0.36 (CI, − 0.09 to − 0.58), *p* = 0.01. d, rho = − 0.42 (CI, − 0.15 to − 0.63), *p* = 0.003

There was significant correlation between the change in 25(OH)D2 level and change in 25(OH)D3 level at day-28 (rho = − 0.79 (CI -0.66 to − 0.88), *p* < 0.001) (Fig. 5a) and day-56 (rho = − 0.69 (CI,

-0.51 to − 0.81), *p* < 0.001). However, there was no significant correlation between baseline total 25(OH)D level and D2-associated change in 25(OH)D3 level at day-28 (rho = − 0.17 (CI, 0.12 to − 0.43), *p* = 0.31) (Fig. 5b) or day-56 (rho = − 0.10 (CI, 0.19 to − 0.37), *p* = 0.50).

### D3-associated decline in 25(OH)D2 level (study-2)

Loading with a single oral dose of 50,000 IU D2 raised 25(OH)D2 mean level to 39.6 (14.1) and 37.5 (15.5) nmol/L in the D3 and placebo arms of study-2, respectively (Table 1)**.** As shown in Fig. 2c, the subsequent administration of a single oral dose of 50,000 IU D3 increased 25(OH)D3 from 45.4 (15.6) nmol/L at day zero to 62.6 (20.8) nmol/L at day-14, which then declined to 53.8 (20.1) nmol/L at day-56. There was little change (45.2 (17.3), 46.6 (23.0), and 42.2 (15.3) nmol/L at days zero, 14, and 56, respectively) in mean 25(OH)D3 level in the placebo arm. In the D3 arm, mean 25(OH)D2 level declined from 39.6 (14.1) nmol/L at day zero to 29.1 (11.0) nmol/L at day-14 and 18.4 (11.5) nmol/L at day-56. In contrast, in the placebo arm, there was further increase in 25(OH)D2 level to 43.9 (21.2) nmol/L at day-14 followed by a progressive decline to 19.0 (8.3) nmol/L at day-56, so that the difference between the two arms was essentially lost (Fig. 2d).

As shown in Fig. 3c, the decline in 25(OH)D2 level in the D3 arm at day-28 (17.5 (7.9) nmol/L) was significantly different from the observed decline in the placebo arm (7.1 (8.0) nmol/L) with an adjusted mean difference of 9.8 (95% confidence interval CI (CI), 5.2 to 14.4) nmol/L, *p* < 0.001. However, the corresponding adjusted mean difference at day-56 was only 1.7 (CI, − 7.6 to 11.1) nmol/L, *p* = 0.71 (Fig. 3d)**.** Of note, the difference between the D3 and placebo arms in 25(OH)D2 level was larger at day-14 than day-28.

Figure 4 shows 25(OH)D2 AUC_28_ and AUC_56_ in the placebo and D3 and arms. Mean 25(OH)D2 AUC_28_ was 1116.8 (420.0) and 863.5 (270.6) nmol.d/L in the placebo and D3 arms, respectively, with a significant adjusted mean difference of 282.2 (CI, 111.2 to 453.3) nmol.hr./L, *p* = 0.002 (Fig. 4c).The corresponding adjusted mean difference at day-56 was 431.2 (CI, 179.3 to 683.2) nmol.hr./L, *p* = 0.001 (Fig. 4d)**.** Compared to placebo, 25(OH)D2 AUC_28_ and AUC_56_ were reduced by 25 and 23%, respectively.

There was significant correlation between the change in 25(OH)D3 level and change in 25(OH)D2 level at day-28 (rho = − 0.36 (CI, − 0.09 to − 0.58), *p* = 0.01) (Fig. 5c) but not day-56 (rho = − 0.03(CI, 0.26 to − 0.31), *p* = 0.84). In addition, there was significant correlation between baseline total 25(OH)D level and the D3-associated change in 25(OH)D2 level at day-28 (rho = − 0.42(CI, − 0.15 to − 0.63), *p* = 0.003) (Fig. 5d) and day-56 (rho = − 0.32(CI, − 0.04 to − 0.55), *p* = 0.03).

## Discussion

We postulated that the frequently-observed, D2 treatment-associated decrease in 25(OH)D3 level may be related to an increase in total 25(OH)D level rather than being specific to D2 treatment, and thus there would be a similar D3 treatment-associated decrease in 25(OH)D2 level. We conducted two randomized placebo-controlled studies. In study-1, we examined the effect of D2 on 25(OH)D3 level. In study-2, we examined the effect of D3 on 25(OH)D2 level. Primary outcome measures were day-28 change in 25(OH)D2 and 25(OH)D3 levels, adjusted for baseline levels. Secondary predetermined outcome measures were the corresponding changes at day-56, correlation between day-28 changes in 25(OH)D2 and 25(OH)D3 levels, and correlation between day-28 changes in 25(OH)D3 (study-1) and 25(OH)D2 (study-2) levels and baseline total 25(OH)D level. We found that: 1) Compared to placebo, a single oral dose of 50,000 IU D2 significantly reduced 25(OH)D3 by 13.2 nmol/L at day-28 and by 10.8 nmol/L at day-56. 2) Compared to placebo, a single oral dose of 50,000 IU D3 significantly reduced 25(OH)D2 by 9.8 nmol/L at day-28 and insignificantly by 1.7 nmol/L at day-56. 3) There was significant negative correlation between changes in 25(OH)D2 and 25(OH)D3 levels in both studies. 4) There was significant correlation between the change in 25(OH)D2 level and baseline total 25(OH)D level in study-2. 5) Finally, compared to placebo, 25(OH)D3 AUC_28_ and AUC_56_ were significantly reduced by 18 and 21%, respectively, in study-1, and 25(OH)D2 AUC_28_ and AUC_56_ were significantly reduced by 25 and 23%, respectively, in study-2.

### D2-associated decline in 25(OH)D3 level

Most [14–18] but not all [2] studies have reported that D2 treatment is associated with a reduction in 25(OH)D3 level. Further, a significant decrease in 1,25(OH)_2_D3 level was seen in response to 1000 IU D2 daily for 11 weeks [19] and 4000 IU D2 daily for 8 weeks [20]. Recently, we observed that 50,000 IU D2, given as a single or divided (daily or 2-weekly) dose, was associated with a significant decrease in 25(OH)D3 level of 10.6 nmol/L at day-28 [10]. Consistently, in the current study (study-1), compared to placebo, a single oral dose of 50,000 IU D2 was associated with a significant decrease in 25(OH)D3 level of 13.2 nmol/L at day-28 and 10.8 nmol/L at day-56 and a significant decrease in 25(OH)D3 AUC_28_ and 25(OH)D3 AUC_56_ of 18 and 21%, respectively.

### D3-associated decline in 25(OH)D2 level

The effect of D3 treatment on 25(OH)D2 levels has not been systematically studied before. However, in one subject with measurable 25(OH)D2 level, 25,000 IU D3 2-weekly over 140 days was associated with about 16 nmol/L decrease in 25(OH)D2 level [10], in a randomized placebo-controlled trial on cardiovascular risk outcome in healthy postmenopausal women, 400 and 1000 IU D3 daily for one year were associated with a decrease in 25(OH)D2 level [17], and in a crossover study on cows, 10,000,000 IU D3 significantly reduced 25(OH)D2 response to 10,000,000 IU D2 [28].

A D3-assocaited decrease in 25(OH)D2 level would be difficult to observe without clearly measurable 25(OH)D2 level. Therefore, in the current study (study-2), we pre-loaded our participants with a single oral dose of 50,000 IU D2, 4 days before randomization to 50,000 IU D3 or placebo. Under this setting, D3 was associated with a significant decrease of 9.8 nmol/L in 25(OH)D2 level at day-28 and a significant decrease of 23 and 25% in 25(OH)D2 AUC_28_ and 25(OH)D2 AUC_56_, respectively. The data strongly support the hypothesis that D2 treatment-associated decrease in 25(OH)D3 level is a reciprocal phenomenon, i.e., D3 treatment is also associated with a decrease in 25(OH)D2 level.

Of note, the significant difference between the placebo and D2 arms in 25(OH)D3 level in study-1 persisted to study conclusion at day-56. In contrast, the difference between the placebo and D3 arms in 25(OH)D2 level in study-2 gradually decreased and essentially disappeared by day-58 (Fig. 2)**.** This is apparently related to different time-courses of 25(OH)D3 and 25(OH)D2 levels in the placebo arms in the two studies; whereas 25(OH)D3 level did not change between day-28 and day-56, 25(OH)D2 level progressively declined. Consistently, the slope of the decrease in 25(OH)D2 level in the D2 arm of study-1 (from 39.8 (11.0) to 19.4 (6.9) nmol/L between days 14 and 56, Fig. 2a) was much sharper than the slope of the decrease in 25(OH)D3 level in the D3 arm of study-2 (from 62.6 (20.8) to 53.8 (20.1) nmol/L between days 14 and 56, Fig. 2c). The fast spontaneous decline of 25(OH)D2 level may be another factor that may obscure the effect of D3 on 25(OH)D2 level.

### Potential mechanisms underlying the decline in 25(OH)D3 and 25(OH)D2 levels

Our finding that D3 is associated with a decrease in 25(OH)D2 level that is similar in magnitude to the D2-associated decrease in 25(OH)D3 level suggests a common underlying mechanism, such as an increase in total 25(OH)D level. In fact, there were significant correlations between the change in 25(OH)D2 level and the change in 25(OH)D3 level in study-1 and between the change in 25(OH)D3 level and the change in 25(OH)D2 level in study-2. Further, there was significant correlation between D3-associated decline in 25(OH)D2 and baseline total 25(OH)D level in this study and between D2-associated decline in 25(OH)D3 and baseline total 25(OH)D in a previous study [10].

These findings are consistent with previous results [10] and with the observation that there is significant negative association between baseline 25(OH)D level and response to vitamin-D treatment [8–10, 18, 21, 22], which may explain about 20% of response variation [9]. Together with the non-linear response in 25(OH)D level to increasing doses of vitamin-D [23–25], the data suggest a regulatory mechanism [24].

### Relative potency of D2 and D3 in raising 25(OH)D level

If D3-associated decrease in 25(OH)D2 level is similar in magnitude to the D2-associated decrease in 25(OH)D3 level, why would D3 be superior to D2 in raising total 25(OH)D as observed in most [4, 5, 8, 10–13] but not all [2, 6, 7] studies?

D2 and D3 differ in their side chains, which affects their affinity to circulating D binding proteins and the hydroxylases involved in their metabolism [8, 11, 12]. Although hepatic 25-hydroxylase has higher affinity to D3 compared to D2 in vitro [8, 12], D2 may be 25-hydroxylated faster than D3 in vivo [10]; likely due to higher accessibility to extra-vascular tissues because of lower affinity to circulating D binding protein [11, 12]. In the current study, after receiving 50,000 IU D, 25(OH)D2 level increased by around 40 nmol/L at day-14 in the D2 arm of study-1 (and by slightly less amount by day-4 in both arms of study-2). This is compared to around only 17 nmol/L increase in 25(OH)D3 level in the D3 arm of study-2. Nevertheless, as noted previously [5, 10, 13], 25(OH)D2 has shorter plasma half-life (compare the decrease after day-14 in 25(OH)D2 level in the D2 arm of study-1 and in both arms of study-2 to the decline in 25(OH)D3 level in the D3 arm of study-2), likely due to higher accessibility to extra-vascular tissues, selective fat tissue storage and recycling of D3 [4], as well as direct 24-hydroxylation of D2 [12]. Thus the superiority of D3 over D2 in raising total 25(OH)D level appears to be time-dependent.

Interestingly, D3 superiority was seen in studies that used large doses but not in most studies that used small doses [1, 9, 11]. In fact, using equivalent total doses, although D3 2-weekly and D3 4-weekly regimens were superior to the corresponding D2 regimens, D2 daily regimen was superior to D3 daily regimen in raising total 25(OH)D level [10]. It is possible that daily dosing may mask the shorter half-life of 25(OH)D2. Thus, in addition to baseline level [8–10, 18, 21, 22], body weight [9, 10, 21, 29–31], sex hormones [9, 10, 31], meal content [32], duration of follow up [10], and non-linear dose-response curve [23–25], dosing strategy should be considered in evaluating response to D2 and D3 supplements.

### Limitations

The interpretation of the results of this study may be limited by its sample size, withdrawal/exclusion rate, variation between screening and baseline 25(OH)D2 and 25(OH)D3 levels, and study-unrelated exposure to D2 and D3. Although the sample was adequately powered for the primary outcome (difference between the two arms in each study), it was not adequately powered for the secondary outcomes or for comparing the active intervention-placebo differences of the two studies. The withdrawal/exclusion rate was highest in the D3 arm of study-2. However, overall, only four of the 100 participants withdrew/were excluded from some or all analyses; and thus withdrawal/exclusion would not be expected to affect the main findings of the study. Baseline 25(OH)D2 and 25(OH)D3 levels in some participants were not within the enrolment criteria, likely due to the time lapse between screening and enrolment, which may have allowed some decline in 25(OH)D2 and 25(OH)D3 levels as well as vitamin D ingestion or sun exposure, possibly induced by knowing screening results. Although this indicates that baseline levels rather than enrolment criteria should be used to characterize our study sample, it would not be expected to affect the main conclusions of the study. Finally, as inferred from measurable D2 and D3 levels, 12% of study-1 participants had study-unrelated exposure to D3, and about one quarter and one third of study-2 participants had study-unrelated exposure to D2 and D3, respectively (the higher rate in study-2 may be related to participants knowledge of their relatively lower 25(OH)D levels). Nevertheless, the study-unrelated exposure was equally distributed between the two arms of each study and thus would not be expected to alter the main conclusions of the study.

## Conclusions

We conclude that D3 is associated with a decrease in 25(OH)D2 level similar in magnitude to D2-associated decrease in 25(OH)D3 level, suggesting a common underlying mechanism. Since the changes in 25(OH)D2 and 25(OH)D3 levels were correlated with each other and with baseline 25(OH)D level, and given the well-known association between baseline 25(OH)D level and response to D supplement, it is possible that total 25(OH)D level is weakly but rather tightly regulated. Changes in 25(OH)D3 and 25(OH)D2 levels in response to D2 and D3, respectively, could be explored for determining “normal” total 25(OH)D level. D3 appears to be more potent than D2 in raising 25(OH)D level, not because D2 reduces 25(OH)D3 level, since such effect is reciprocal, but because 25(OH)D2 has shorter plasma half-life, which may be overcome by daily supplementation.

## Abbreviations

1,25(OH)_2_D2: 1,25-dihydroxyvitamin D2
1,25(OH)_2_D3: 1,25-dihydroxyvitamin D3
25(OH)D: Total 25-hydroxyvitamin D (25(OH)D2 + 25(OH)D3)
25(OH)D2: 25-hydroxyvitamin D2
25(OH)D3: 25-hydroxyvitamin D3
AUC_28_: Area-under-the-curve from baseline to day 28
AUC_56_: Area-under-the-curve from baseline to day 56
BMI: Body mass index
CI: Confidence interval
D: Vitamin-D
D2: Vitamin-D2 or ergocalciferol
D3: Vitamin-D3 or cholecalciferol
HPLC: High performance liquid chromatography
KFSH&RC: King Faisal Specialist Hospital and Research Center
